# An unusually high upper thermal acclimation potential for rainbow trout

**DOI:** 10.1093/conphys/coab101

**Published:** 2022-01-15

**Authors:** Olivia A Adams, Yangfan Zhang, Matthew H Gilbert, Craig S Lawrence, Michael Snow, Anthony P Farrell

**Affiliations:** Department of Zoology, The University of British Columbia, Vancouver, British Columbia, Canada; Faculty of Land and Food Systems, The University of British Columbia, Vancouver, British Columbia, Canada; Museum of Comparative Zoology, Department of Organismic and Evolutionary Biology, Harvard University, Cambridge, Massachusetts, United States; Department of Zoology, The University of British Columbia, Vancouver, British Columbia, Canada; Faculty of Science, School of Agriculture and Environment, The University of Western Australia, Perth, Western Australia, Australia; Aquatic Life Industries, Perth, Western Australia, Australia; Faculty of Land and Food Systems, The University of British Columbia, Vancouver, British Columbia, Canada

**Keywords:** standing variation, hypoxia, heart rate, growth, digestion, aerobic capacity

## Abstract

Thermal acclimation, a compensatory physiological response, is central to species survival especially during the current era of global warming. By providing the most comprehensive assessment to date for the cardiorespiratory phenotype of rainbow trout (*Oncorhynchus mykiss*) at six acclimation temperatures from 15°C to 25°C, we tested the hypothesis that, compared with other strains of rainbow trout, an Australian H-strain of rainbow trout has been selectively inbred to have an unusually high and broad thermal acclimation potential. Using a field setting at the breeding hatchery in Western Australia, thermal performance curves were generated for a warm-adapted H-strain by measuring growth, feed conversion efficiency, specific dynamic action, whole-animal oxygen uptake (*Ṁ*O_2_) during normoxia and hypoxia, the critical maximum temperature and the electrocardiographic response to acute warming. Appreciable growth and aerobic capacity were possible up to 23°C. However, growth fell off drastically at 25°C in concert with increases in the time required to digest a meal, its total oxygen cost and its peak *Ṁ*O_2_. The upper thermal tipping points for appetite and food conversion efficiency corresponded with a decrease in the ability to increase heart rate during warming and an increase in the cost to digest a meal. Also, comparison of upper thermal tipping points provides compelling evidence that limitations to increasing heart rate during acute warming occurred well below the critical thermal maximum (CT_max_) and that the faltering ability of the heart to deliver oxygen at different acclimation temperatures is not reliably predicted by CT_max_ for the H-strain of rainbow trout. We, therefore, reasoned the remarkably high thermal acclimation potential revealed here for the Australian H-strain of rainbow trout reflected the existing genetic variation within the founder Californian population, which was then subjected to selective inbreeding in association with severe heat challenges. This is an encouraging discovery for those with conservation concerns for rainbow trout and other fish species. Indeed, those trying to predict the impact of global warming should more fully consider the possibility that the standing intra-specific genetic variation within a fish species could provide a high thermal acclimation potential, similar to that shown here for rainbow trout.

## Teaser

We address a crucial question relevant to global climate change and of general interest to ecological and conservation physiologists: does a fish species normally regarded as a cold-loving species have sufficient standing intra-specific variation to form a warm-tolerant population that could perhaps thrive in a warmer world?

## Introduction

Fishes, being the most specious vertebrate taxa, have a diverse physiology that has allowed them to exploit almost every aquatic habit, including those with water temperatures ranging from −1.9°C to over 40.0°C (Davenport and Sayer, 1993). All the same, certain species are being displaced from their historical biogeographic ranges during the current era of global warming because thermal performance ranges of fishes are species specific and more biogeographic shifts are predicted for a warmer future (Cheung *et al.,* 2006; Pörtner and Farrell, 2008; Pereira *et al.,* 2010). Specifically, the geographic ranges of fishes occupying narrow and warm thermal niches (i.e. warm-water thermal specialists) have contracted or shifted as a result of climate warming and other similar species are at risk of displacement as our climate continues to warm. Thus, global warming is clearly a conservation concern. However, the models predicting these biogeographic shifts rarely consider the potential for a fish species to acclimate or adapt to new thermal conditions—possibilities that are becoming evident among fishes as evidenced by intra-specific local adaptations of thermal tolerance and thermal performance (McKenzie *et al*., 2021).

Good examples of intra-specific local adaptations of thermal tolerance and thermal performance include killifish (*Fundulidae* spp.) populations along the east coast of North America (Fangue *et al*., 2006), Lake Magadi tilapia (*Tilapia grahami*) in Africa (Reite *et al.,* 1974), Atlantic cod (*Gadus morhua*) populations in the North Atlantic Ocean (Grabowski *et al.,* 2009), Chinese minnow (*Rhynchocypris oxycephalus*) populations in Northeast coast of China (Yu *et al.,* 2018), sockeye salmon (*Oncorhynchus nerka*) populations in the Pacific Northwest (Eliason *et al.,* 2011), perch (*Perca fluviatilis*) populations in Sweden (Sandblom *et al.,* 2016) and redband trout (*Oncorhynchus mykiss gairdneri*) populations in Idaho (Rodnick *et al.,* 2004; Chen *et al.,* 2018). Nevertheless, intra-specific variability in thermal tolerance is not evident in all fish species. For example, a northern and a southern population of Atlantic salmon (*Salmo salar*) did not show local adaptation of their heat tolerance (Anttila *et al.,* 2014), but instead the two populations possessed a considerable potential for thermal acclimation (i.e. phenotypic thermal plasticity), the ability to maintain or improve performance after prolonged exposure to a new temperature. Consequently, population-based modelling will need reliable information on local intra-specific adaptation and the acclimation potential of their upper thermal ceiling (Sandblom *et al.,* 2016). Indeed, Lefevre *et al.* (2017) have already suggested that reliable predictions about fish populations under climate change will need to consider the underlying physiological mechanisms, some of which we sought here for temperature acclimation.

In the present study, we focussed on the thermal acclimation potential of rainbow trout (*O. mykiss*) in part because of its successful introductions from the endemic habitat in the Pacific Northwest onto every continent, except Antarctica. Yet, rainbow trout are considered a ‘cold-water’ species, one that prefers temperatures <20°C (MacCrimmon, 1971). Indeed, historically, rainbow trout spread southwards in the Pacific Northwest as a post-glacial colonizer ~10 000 years ago. Furthermore, the US Environmental Protection Agency (USEPA) recommends a 7-day average daily mean maximum of 18°C for the management of their watersheds in the US Pacific Northwest (US Environmental Protection Agency, 2003). Nevertheless, population differences exist for the thermal performance of rainbow trout (Myrick and Cech, 2000, 2004; Rodnick *et al.,* 2004; Verhille *et al.,* 2016) and warm acclimation can increase their critical thermal maximum (CT_max_; Becker and Wolford, 1980; Myrick and Cech, 2000).

Of interest to us was the H-strain of rainbow trout, which has been genetically isolated and inbred for stocking natural lakes for over 20 generations at the Pemberton Freshwater Research Centre (PFRC), a hatchery in arid Western Australia. Over this time, the genetic diversity of the H-strain became reduced compared with the founder population that originated from Sonoma Creek in the San Francisco Bay area, USA, likely as a result of inbreeding and periodic thermal selection events over the decades (i.e. natural summer temperature extremes >27°C in a reduced water supply from the Lefroy Brook that caused large mortality events among the fish stock: 66% in 1961 and >98% in 1970) (Ward *et al.,* 2003; Molony *et al.,* 2004). When acclimated to only 15°C, the acute thermal tolerance of the H-strain is already impressive compared with Pacific Northwest populations. For example, their critical thermal maximum (CT_max_) is an impressive 29°C and when acutely warmed to 25°C, they maintain 43% of their maximum aerobic capacity (Chen *et al.,* 2015). However, no one has tested the hypothesis that warm acclimation of this H-strain of rainbow trout can further improve their upper thermal performance.

To test this hypothesis and to seek mechanistic insights into thermal performance, we comprehensively assessed the cardiorespiratory phenotype of the H-strain of rainbow trout at the PFRC field setting. By using 2°C increments for the six acclimation temperatures (from 15 to 25°C), we could more finely resolve thermal optima compared with 3–5°C temperature increments (e.g. Christensen *et al.,* 2020) that are more typically used (Farrell, 2016) and focus on upper tipping points of the thermal performance curves. Specifically, we measured growth, whole-animal aerobic capacity, the energetic cost to digest a meal and their CT_max_, as well as hypoxia tolerance (because hypoxia tolerance and CT_max_ have been previously linked among other strains of rainbow trout in the Pacific Northwest; Zhang *et al.,* 2018). At each acclimation temperature, we also measured the response of maximum heart rate (f_Hmax_) to acute warming as an increase in heart rate (f_H_) is the primary cardiac response to acute warming and warm acclimation can increase the peak f_Hmax_ reached with acute warming (Eliason and Anttila, 2017; Farrell, 2016; Vornanen, 2017).

## Methods

The broodstock of H-strain rainbow trout has been maintained for almost 50 years at the PFRC (Molony, 2001), which is at a remote location south of Perth, Western Australia. We used fish from the general hatchery population (approximately 100 parents) that were bred in spring 2018 and then raised for 9 months in aerated ponds that received water from the Big Brook dam on the Lefroy Brook, Western Australia. During rearing, water temperature fluctuated daily (solar effect) and seasonally according to ambient temperature fluctuations. Experimental procedures and protocols were approved by the University of British Columbia Animal Care Committee (A18-0340).

For the present experiment 3000 rainbow trout were transferred into indoor, 250-l fibreglass holding tanks (50–80 fish per tank), where they were held before any experimentation for a minimum of 4 weeks at one of the six acclimation temperatures (15, 17, 19, 21, 23 or 25°C) with a 12:12 h light/dark cycle. The water temperatures were kept constant and did not incorporate variation that would occur in the natural water supply from the Big Brook Dam. Each tank contained a biological filter to remove particulates and an immersion heater to raise the water temperature above that of the ambient receiving water, as needed.

Experimentation started in February 2019 and lasted through to June 2019. A growth trial was performed in triplicate at each acclimation temperature (50 fish in each tank; 18 tanks in total). A further 80 fish were held at each acclimation temperature in additional, separate tanks for exclusive use with the physiological tests. The periodic removal of fish for physiological measurements, therefore, did not disturb the growth trial. Each fish was used for one experiment at each acclimation temperature except that six fish were reused after the aerobic scope tests to examine the response of *f*_Hmax_ to acute warming. Mortality was monitored daily in each tank. Fish mass is reported in Supplemental Table S1.

### Growth trial

Fish were fed to satiation with commercial freshwater trout feed (Skretting, Cambridge, Tasmania, Australia) three times daily, except on the day preceding and the day of weighing for growth measurements. Individual fish mass and length were measured at weeks 0, 2 and 4 of the growth trials. The amount of food added to each tank was recorded daily, which allowed a calculation of food conversion efficiency for the total mass of fish in the tank (i.e. food conversion efficiency was measured in triplicate at the tank level for each acclimation temperature).

### Respirometry trials to determine the respiratory phenotype

Established protocols and analytical procedures (Chabot *et al.,* 2016b; Zhang *et al.,* 2016; Zhang *et al.,* 2017) were used to monitor oxygen uptake (*Ṁ*O_2_) of individual fish at their acclimation temperature over a period of 2 days. Each trial monitored eight fish simultaneously and separately in eight respirometers (760 ml), which were all submerged in a 200-l water reservoir supplied with a recirculating chiller and immersion heaters to maintain water at the required acclimation temperature. A circulation loop for each respirometer continuously mixed the water inside respirometry chamber and fibre optic oxygen probe (Firesting O_2_, PyroScience GmbH, Aachen, Germany) contained in this loop recorded the water oxygen level (as % of air saturation; % air sat.) inside the respirometer every 1 s. The optodes were calibrated using aerated water (100% air saturation) and nitrogen bubbled water containing sodium sulphite (0% saturation). Water oxygen levels were converted to water PO_2_ (mm Hg) and dissolved oxygen concentration (mg O_2_ l^−1^) for data analysis, as needed.

All respirometers had computer-controlled flush pumps (Compact Pump 1000, Eheim, Germany) and relays (AquaResp, University of Copenhagen, Denmark) that controlled the intermittent replenishment of the respirometer with fresh water between each *Ṁ*O_2_ measurement period. Each 10-min cycle consisted of a 60-s flush period, a 60-s stabilization period and a 480-s period when *Ṁ*O_2_ was measured. During this *Ṁ*O_2_ measurement period, *Ṁ*O_2_ was calculated as the rate of the decrease in dissolved oxygen over time (Chabot *et al.,* 2016a). A UV-sterilizer light was placed in the water reservoir to reduce microbial growth. All *Ṁ*O_2_ measurements were corrected for the background *Ṁ*O_2_, which was measured in each respirometer without a fish (for 30 min) both before and immediately after every trial. The entire respirometry system was thoroughly disinfected with 10% bleach for 1 h and thoroughly cleaned between trials.

The respiratory phenotype of individual rainbow trout was measured for 16 fish per acclimation temperature, i.e. two trials with 8 fish for each acclimation temperature. Each trial was preceded by a 48-h fast and a measurement of body mass (Table S1) before securing a fish inside a respirometer typically between 12.00 h and 14.00 h. Each fish was individually agitated inside the respirometer (to exhaustion but no longer than 10 min) to elicit a maximum rate of oxygen uptake (*Ṁ*O_2max_), which was assigned to the highest *Ṁ*O_2_ value observed while the fish recovered (Zhang *et al.,* 2020). Each fish was then left undisturbed for 48 h, during which time *Ṁ*O_2_ was continuously monitored (~150 *Ṁ*O_2_ values were generated per fish). From these *Ṁ*O_2_ measurements, standard metabolic rate (SMR) was estimated by applying a 20th quantile algorithm (q0.2; Chabot *et al.,* 2016b), while absolute aerobic scope (AAS) was calculated as the difference between *Ṁ*O_2max_ and SMR (Fry, 1971; Claireaux *et al.,* 2005) and factorial aerobic scope (FAS) was calculated as the quotient of *Ṁ*O_2max_ to SMR (Clark *et al.,* 2005). The respirometry trial ended with a hypoxia challenge test that lasted 2–3 h, during which nitrogen gas was bubbled through a ceramic micro-bubble diffuser into the water reservoir such that the % air saturation progressively decreased by 0.3% min^−1^ until the % air saturation was so low that the fish lost its dorsal-ventral equilibrium. This ambient level of % air saturation was recorded and converted to an incipient lethal oxygen concentration (ILOC, mg O_2_ l^−1^) or incipient lethal oxygen partial pressure (ILOP, mmHg). Fish were immediately revived in aerated water. The % water saturation when SMR could not be maintained (either C_crit_, mg O_2_ l^−1^ or P_crit_, mmHg) was interpolated by graphing *Ṁ*O_2_ as a function of % water saturation during progressive hypoxia (Schurmann and Steffensen, 1997; Claireaux and Chabot, 2016).

### Individual feeding trials to determine specific dynamic action

Fish were individually fed to satiation in the acclimation holding tank, weighed and transferred immediately into a respirometer (as described above) at their acclimation temperature with minimal air exposure (maximum 20 s). Thus, continuous *Ṁ*O_2_ measurements started within 20 min after feeding ceased and continued for 48–72 h depending on when *Ṁ*O_2_ returned to SMR (q0.2 of *Ṁ*O_2_ recordings) for 10–15 measurements per acclimation temperature (see Table S1 for body mass). The SMR of fish in the feeding trial turned out to be similar (*t*_10_ = 0.62, *P* = 0.55) to that of fish in the respirometry trials described above. Fish were euthanized at the end of the specific dynamic action (SDA) trial (80 mg l^−1^ MS222 buffered with 160 mg l^−1^ NaHCO_3_) before being weighed. Analysis of SDA between the start of feeding and the return to SMR (SDA _duration_, h) started with an inspection of individual *Ṁ*O_2_ traces to ensure adherence to established analysis criteria (Chabot *et al.,* 2016a). Short periods of activity were smoothed with a moving average algorithm, and a non-parametric quantile regression algorithm was used to describe the SDA curve. The total amount of oxygen consumed during SDA (SDA magnitude, mg O_2_ kg^−1^) was determined by an integral of the area below the fitted SDA curve above the SMR and the peak *Ṁ*O_2_ on the fitted curve was assigned as SDA_peak_ (mg O_2_ h^−1^ kg^−1^).

### **Response to acute warming: CT**_**max**_  **and maximum heart rate (*f***_**Hmax**_**) measurements**

CT_max_ was measured using an established methodology (Beitinger *et al.,* 2000) by rapidly transferring 10 fish after a 24-h fast into a single 200-l aerated tank at 12°C. They habituated to the tank for 1 h before temperature was incrementally increased at a rate of 0.3°C min^−1^ to 22°C using two heating rods (100 W Titanium Heater, Aquatop, Brea, CA, USA). Subsequently the heating rate was reduced to 0.1°C min^−1^, until a temperature was reached that caused individual fish to lose equilibrium (their CT_max_), whereupon the individual was immediately removed and revived in a recovery bath at their acclimation temperature.

Similarly, an established methodology (Casselman *et al.,* 2012) was used to measure *f*_Hmax_ during an acute, incremental warming protocol. From each respirometry trial, 6 of the 10 fish were placed in a recovery tank for 4 h, using a total of 12 fish per acclimation temperature. Briefly, a fish was anaesthetized (80 mg l^−1^ MS222 buffered with 160 mg l^−1^ NaHCO_3_) and transferred to a sling immersed in a 12°C water bath where its gills were continuously irrigated with recirculating water containing a maintenance dose of anaesthetic (65 mg l^−1^ MS222 buffered with 130 mg l^−1^ NaHCO_3_). An electrocardiograph (ECG) was recorded on-line using two stainless steel electrodes, one inserted below the muscle layer near the heart and the second below the pelvic fin as a reference. The ECG signal was amplified and digitized (Animal Bio Amp and a Powerlab 8/30, ADInstruments Inc., Bella Vista, NSW, Australia), and *f*_H_ was extracted from the ECG signal using Labchart software (ADInstruments). To obtain *f*_Hmax_, vagal tone to the heart was blocked with an intra-peritoneal injection of atropine sulphate (1.8 mg kg^−1^) and cardiac adrenergic β-receptors were maximally stimulated with an injection of isoproterenol (6 μg μg kg^−1^) (Sigma Chemicals, Perth, Western Australia). The incremental heating protocol began after a 30-min period at 12°C, which provided time for the pharmaceutical agents to have their effects and ample time for the fish to reach thermal equilibrium with the ambient water temperature. By incrementally heating the water bath by 1°C every 6 min, we ensured that *f*_Hmax_ had stabilized at each temperature increment. Warming continued until a temperature was reached when *f*_Hmax_ peaked (T_peak_). Further incremental warming was terminated at the temperature that first generated a cardiac arrhythmia (T_arr_), at which point the fish was euthanizing with a lethal dose of anaesthetic. The temperature quotient (Q_10_) for *f*_Hmax_ during acute warming (Casselman *et al.,* 2012) was calculated as the ratio of *f*_Hmax_ values for 15°C and 25°C at each acclimation temperature.

### Statistical analysis

Data analysis was conducted in R (v.3.6.2, R Core Team, Austria) and Prism (v.8, GraphPad Software, USA). All values are presented as means ± standard error of the mean (sem) with statistical significances assigned when α = 0.05. For the growth study, a mixed effects analysis was performed on fish mass and length (tank ID and time as fixed effects, acclimation as a random effect, mass and length were fit with a Gompertz growth non-linear regression with a least square fit) while feeding was examined with a two-way analysis of variance (ANOVA) followed by a Tukey’s post hoc-test (Table S2). Differences in acclimation temperatures were examined for each variable using a one-way ANOVA followed by a Tukey’s post-hoc test (Table S3). To account for allometric scaling, oxygen consumption data (SMR, *Ṁ*O_2max_, AAS) were adjusted to the average body mass (16.23 g) by summing the residuals for the linear regression between mass and oxygen consumption with the predicted value at the average body mass (16.23 g). This method was applied to oxygen consumption data for SDA values (SMR, peak and net peak; average body mass 16.17 g). Regression analysis was performed between the performance parameter and acclimation temperature to determine the line of best fit (quadratic polynomial for SMR and ILOS, Gaussian function for *Ṁ*O_2max_, AAS and FAS). Differences in CT_max_ and T_arr_ were tested using a two-way ANOVA followed by a Tukey’s post-hoc test (Table S2), while T_peak_, peak *f*_Hmax_ and *f*_Hmax_ at 15°C were tested using a one-way ANOVA followed by a Tukey’s post-hoc test (Table S3). A Pearson’s correlation coefficient was determined for the relationship between individual *f*_Hmax_ and individual *Ṁ*O_2max_ values at each acclimation temperature.

## Results

**Figure 1:**
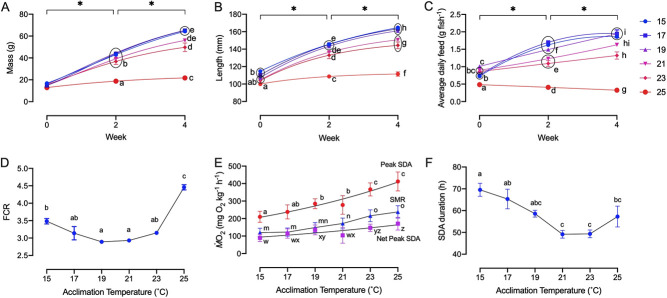
Mass, length, average daily feed, food conversion ratio (FCR), specific dynamic action (SDA) duration and peak SDA, standard metabolic rate (SMR) and net peak SDA values for PFRC H-strain rainbow trout (*O. mykiss*). (**A**) Body mass (g), (**B**) fish length (mm) and (**C**) average daily feed ration (total per tank and averaged to number of rainbow trout in tank and per day) for each temperature acclimation group held in triplicate tanks (*n* = 3) and measured at weeks 0, 2 and 4. (**D**) FCR of rainbow trout as function of acclimation temperature (*n* = 3). (**E**) Peak SDA, measured as the highest oxygen uptake rate (ṀO2) value following feeding, SMR, measured as ṀO2 and net peak SDA measured as the difference between peak SDA and SMR, all presented as a function of acclimation temperature (*n* = 12–16). (**F**) SDA duration measured as a function of acclimation temperature (*n* = 12–16). Data points not sharing letters indicate significant differences between acclimation groups for that week. Asterisk indicates significant differences of all acclimation groups between two consecutive sampling time points. All values are presented as means ± sem. Mass, length and feed were assessed using a linear mixed effects model while FCR, SDA duration, peak SDA, SMR and net peak SDA were assessed using a one-way ANOVA with Tukey’s post-hoc tests.

During the 4-week growth trial, fish gained body mass and length at all six acclimation temperatures, with both metrics increasing significantly by week 2 (F_1.3, 14.9_ = 3690, *P* < 0.0001 and F_1.2, 14.2_ = 4249, *P* < 0.0001, respectively; Fig. 1A). By week 4, however, significant differences in growth were evident among the acclimation temperatures (F_5,11_ = 144.6, *P* < 0.0001). While body mass increased by 3.1- to 4.4-fold after 4 weeks at acclimation temperatures from 15°C to 23°C, the increase in body mass was only 1.7-fold at 25°C (Fig. 1A, B).

Appetite increased as fish grew in size as evidenced by an increase in total daily feed intake over time at acclimation temperatures from 15°C to 23°C (Fig. 1C). However, appetite was significantly suppressed at 25°C for the entire growth trial. The food conversion ratio (FCR; the inverse of the efficiency of feed utilization) was highest (by 30–50%) for the 23°C-acclimated fish compared with the other acclimation temperatures (Fig. 1D). Thus, the significantly lower appetite of the 23°C-acclimated fish (Fig. 1C) was somewhat offset in terms of growth by a low FCR, which was similar for acclimation temperatures between 17°C and 23°C (Fig. 1D). Consequently, peak growth performance occurred over the acclimation temperature range of 17–23°C.

The feeding energetics trial measured the oxygen cost (*Ṁ*O_2_) of digesting a meal (the SDA), which reached a peak value (SDA_peak_) and then decline towards the post-prandial SMR that was also determined. Both SDA_peak_ and post-prandial SMR increased significantly as a function of acclimation temperature (F_5,71_ ≥ 59.8, *P* < 0.0001), both peaking at an acclimation temperature of 25°C (F_5,71_ = 13.08; Fig. 1E). Furthermore, net SDA_peak_ during digestion (the difference between SDA_peak_ and post-prandial SMR) increased significantly with acclimation temperature (*P* ≤ 0.042; Fig. 1E), more than doubled, from 87 mg O_2_ kg^−1^ h^−1^ at 15°C to 183 mg O_2_ kg^−1^ h^−1^ at 25°C. Thus, the allocation of oxygen specifically for digestion increased significantly with temperature acclimation up to 25°C. SDA duration also varied significantly with acclimation temperature, but decreased significantly with acclimation temperature (F_5,71_ = 7.9,*P* < 0.0001; Fig. 1F), perhaps in part because appetite decreased at the warmest acclimation temperatures (F_5,36_ = 168.1, *P* < 0.0001; Fig. 1C). As a result, the total oxygen cost of digesting a meal (SDA magnitude) was lowest at 21°C and highest at 25°C (F_5,71_ = 5.4, *P* < 0.0001; Fig. 1C) despite the much lower appetite of the 25°C-acclimated fish (Fig. 1).

**Figure 2:**
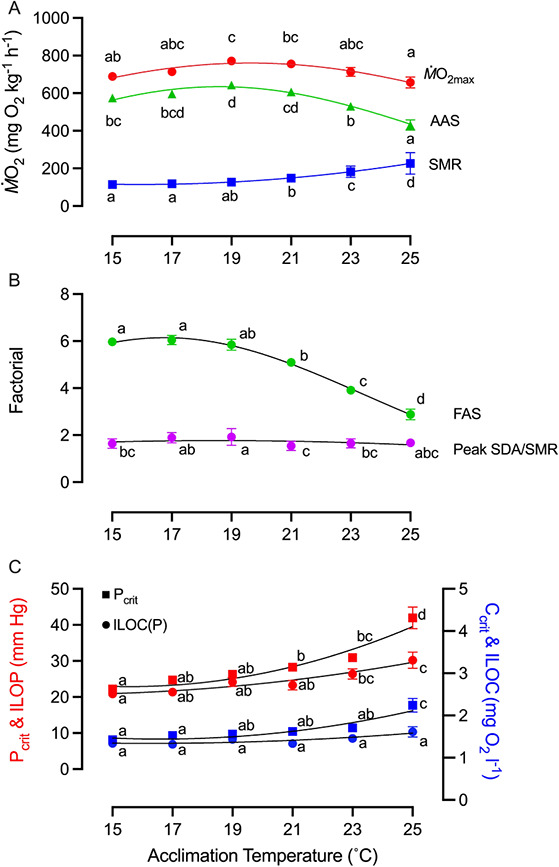
Standard metabolic rate (SMR), maximum oxygen uptake (*Ṁ*O_2max_), absolute aerobic scope (AAS) and factorial aerobic scope (FAS) of PFRC H-strain rainbow trout (*O. mykiss*) as a function of acclimation temperature. (**A**) SMR, *Ṁ*O_2max_ and AAS (difference between *Ṁ*O_2max_ and SMR) as a function of acclimation temperature (*n* = 12–16). SMR (blue square) was fitted with a polynomial quadratic function and one-way ANOVA (Y = 135.0 + 10.5^*^X + 1.35^*^X^2^, R^2^ = 0.67, F = 31.14, *P* < 0.0001), *Ṁ*O_2max_ (red circle) and AAS (green triangle) were fit with a Gaussian distribution curve and one-way ANOVA (Y = 760.2^*^exp(−0.5^*^((X-19.6)/9.89)2), R^2^ = 0.22, F = 4.91, *P* = 0.0006; Y = 634.7^*^exp(−0.5^*^((X-18.6)/7.37)2), R^2^ = 0.42, F = 15.40, *P* < 0.0001). *Ṁ*O_2max_ and SMR values were corrected to mean body mass of acclimation group, presented per unit kg. (**B**) Factorial of *Ṁ*O_2max_/SMR (FAS) and peak SDA/SMR (*n* = 12–16). FAS (green circle) was fitted with a Gaussian distribution curve and one-way ANOVA (Y = 1.9^*^exp(−0.5^*^((X-2.0)/1.7)2), R^2^ = 0.73, F = 43.41, *P* < 0.0001). (C) Critical oxygen level to maintain SMR (P_crit_ or C_crit_) and oxygen level when fish is loss of whole animal equilibrium (ILOP or ILOC), as a function of acclimation temperature. P_crit_ (or C_crit_) and ILOP (or ILOC) are presented as both water PO_2_ (mm Hg; left y-axis) and dissolved oxygen concentration (mg O_2_ l^-1^; right y-axis). Curve was fit with polynomial quadratic function and one-way ANOVA (Pcrit: Y = 26.3 + 1.60^*^X + 0.19^*^X^2^), R^2^ = 0.57, F = 22.19, *P* < 0.0001; Ccrit: Y = 1.54 + 0.062^*^X + 0.0095^*^X^2^), R^2^ = 0.40, F = 11.58, *P* < 0.0001; ILOP: Y = 23.3 + 0.80^*^X + 0.061^*^X^2^), R^2^ = 0.28, F = 6.41, *P* < 0.0001; ILOC: Y = 1.37+ 0.021^*^X + 0.0031^*^X^2^), R^2^ = 0.57, F = 1.91, *P* = 0.10, (n = 12–16). Data points not sharing letters indicate significant differences between acclimation groups for that variable. All values are presented as mean ± sem and were assessed using a one-way ANOVA with Tukey’s post-hoc (α = 0.05).

The respirometry trial, which used unfed fish, provided another independent measurement of SMR (Fig. 2A). The two independent SMR measurements were statistically indistinguishable (*t*_10_ = 0.62, *P* = 0.55). Again, SMR increased significantly with acclimation temperature (F_5,56_ = 26.2, *P* < 0.0001). Maximum oxygen uptake (*Ṁ*O_2max_) peaked at acclimation temperatures between 17°C and 23°C, being statistically indistinguishable at these four acclimation temperatures (F_5, 77_ = 4.91, *P* ≥ 0.19; Fig. 2A), but numerically highest at 19°C (771 ± 16 mg O_2_ kg^−1^ h^−1^). Consequently, the capacity to increase oxygen uptake (AAS = *Ṁ*O_2max_ − SMR) similarly peaked at acclimation temperatures between 17°C and 21°C (F_5,77_ = 2.47, *P* ≥ 0.19; Fig. 2A) and, again, was numerically highest at 19°C (639 ± 18 mg O_2_ kg^−1^ h^−1^). Both *Ṁ*O_2max_ and AAS decreased significantly for the 25°C-acclimation temperature when compared with the 19°C-acclimation temperature (Fig. 2A; *Ṁ*O_2max_: F_5,77_ = 4.9, *P* = 0.0012; AAS: F_5,77_ = 1.6, *P* < 0.0001; Table S3). FAS (the ratio of *Ṁ*O_2max_ and SMR) was greatest at acclimation temperatures between 15°C and 19°C, but decreased significantly at acclimation temperatures ≥21°C (Fig. 2B). Thus, while *Ṁ*O_2_ could be increased beyond SMR by about 6-fold at acclimation temperatures between 15°C and 19°C, the 25°C-acclimated fish could increase in *Ṁ*O_2_ above SMR by less than 4-fold, still sizeable albeit reduced aerobic scope. Notably, when SDA_peak_ was expressed as a ratio of SMR, it was never greater than twice SMR and virtually independent of acclimation temperature (Fig. 2C), suggesting that peak *Ṁ*O_2_ may be mechanistically linked with SMR in a manner largely independent of the acclimation temperature.

Warm acclimation significantly reduced hypoxia tolerance of the H-strain, but the extent of the decrease in hypoxia tolerance depended on the endpoint used and its dimension (Fig. 2C). One hypoxia tolerance endpoint was the threshold water oxygen level to maintain SMR (either P_crit_, mm Hg or C_crit_, mg O_2_ l^−1^; Table S1). Both P_crit_ and C_crit_ increased significantly with acclimation temperature (P_crit_, mm Hg: F_5,69_ = 22.19, *P* < 0.0001; C_crit_, mg O_2_ l^−1^: F_5,69_ = 11.58, *P* < 0.0001). The other hypoxia tolerance endpoint was the threshold water oxygen level for the loss of the righting reflex (either ILOP, mm Hg, or ILOC, mg O_2_ l^−1^). While ILOP increased significantly with acclimation temperature (F_5,73_ = 6.41, *P* < 0.0001), it did so to a lesser degree than P_crit_. Also, ILOC was independent of acclimation temperature (F_5,73_ = 1.91, *P* = 0.10; Fig. 2C). Since ILOP considers the oxygen partial pressure gradient for diffusion at the gills, while ILOC considers the amount of oxygen contained in the water moved over the gills, our results suggest that at 25°C the need for a sufficiently high oxygen partial pressure gradient to oxygenate blood at the gills is perhaps mechanistically more important than the temperature-dependent decrease in water oxygen at this water temperature.

**Figure 3:**
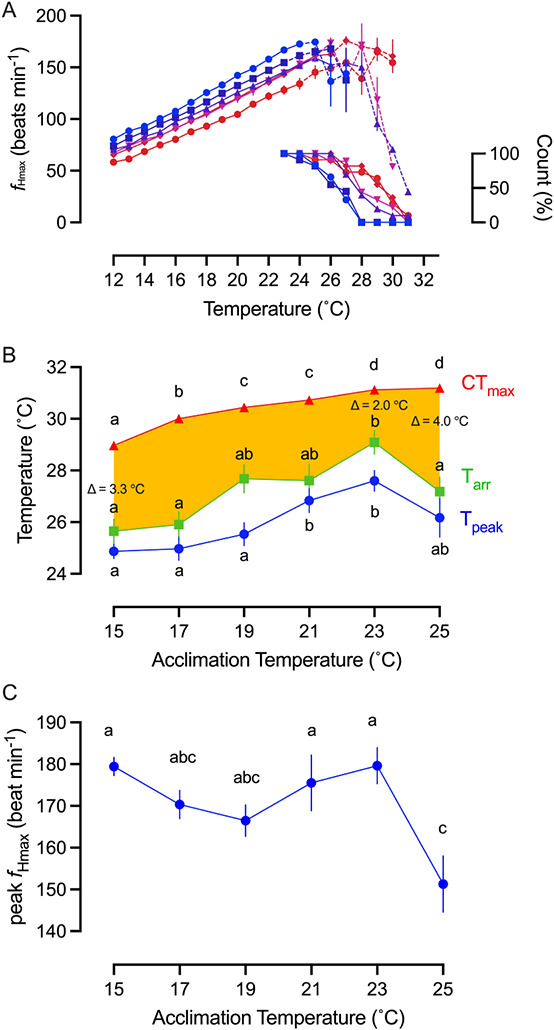
Maximum heart rate (*f*_Hmax_) and critical thermal maximum (CT_max_) in response to acute warming of PFRC H-strain rainbow trout (*O. mykiss*) as a function of acclimation temperature. (**A**) Mean *f*_Hmax_ for each acclimation temperature group during acute warming (*n* = 12). Acclimation temperatures were 15°C (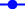), 17°C (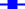), 19°C (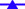), 21°C (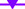), 23°C (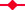) and 25°C (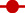). Data points are connected by a solid line provided all fish in an acclimation group retained a rhythmic heartbeat at that temperature. If not a broken line connects the average *f*_Hmax_ for the fish the still retained a rhythmic heartbeat, with the percentage count remaining indicated in the inset. All individual fish had developed an arrhythmic heartbeat by 31°C and by 28°C for those at the two coldest acclimation temperatures. (**B**) Temperature at which each acclimation group reached its CT_max_ (*n* = 10), cardiac arrhythmia temperature (T_arr_) and maximum *f*_Hmax_ (T_peak_). Amber area labels the difference between whole-organism and tissue thermal tolerance and the differences between CT_max_ and T_arr_ at 15°C, 23°C and 25°C acclimation temperature are denoted. Two-way ANOVA performed indicates T_arr_ is significantly different from CT_max_ (F = 213.2, *P* < 0.0001). (**C**) Peak *f*_Hmax_ reached at each acclimation temperature (*n* = 12). All values are presented as means ± sem, except right y-axis in (A) presented as count in %. (sem may be hidden by the symbol). Dissimilar letters represent statistically significant differences among mean values for that variable.

Independent of the acclimation temperature, acute warming always increased *f*_Hmax_ until it reached a peak value (Q_10_ values ranged from 1.70, 1.69, 1.81, 1.92, 1.93 and 1.79 with ascending acclimation temperatures; Fig. 3A). As expected, warm acclimation incrementally reset *f*_Hmax_ to a lower value when a comparison was made at the common test temperature of 15°C (F_5,52_ = 23.11, *P* < 0.0001). For example, at the 15°C test temperature, *f*_Hmax_ was 22 bpm lower for 25°C-acclimated fish compared with 15°C-acclimated fish (58 bpm vs 80 bpm; F_1.83, 32.6_ = 5.96, *P* = 0.0017; Fig. 3A; Table S3). Warm acclimation also significantly increased the temperature for peak *f*_Hmax_ (T_peak_; F_5,56_ = 5.34, *P* = 0.0004). For example, T_peak_ was 2.7°C higher for 23°C-acclimated fish compared with 15°C-acclimated fish (27.6 ± 0.4°C vs 24.9 ± 0.3°C). Thus, warm acclimation, by resetting *f*_Hmax_ and increasing T_peak_, preserved some scope to increase *f*_H_ in response to acute warming. Even so, peak *f*_Hmax_ was independent of acclimation temperature between 15°C and 23°C (ranging from 166 ± 4 to 179 ± 4 bpm; Table S1), and peak *f*_Hmax_ actually decreased significantly for the 25°C-acclimated fish (F_5,56_ = 5.3, *P* ≤ 0.01).

A cardiac arrhythmia was triggered whenever a fish was acutely warmed beyond T_peak_ (i.e. at T_arr_; Fig. 3A inset). Like T_peak_, warm acclimation significantly increased T_arr_ by 3.4°C when 15°C-acclimated fish were compared with 23°C-acclimated fish (25.7 ± 0.5°C vs 29.1 ± 0.5°C; F_5,56_ = 6.29, *P* = 0.0002), but this benefit was lost at a 25°C-acclimation temperature because T_arr_ (27.2 ± 0.6°C) decreased significantly (F_5,56_ = 6.29, *P* = 0.0001; Fig. 3B).

Warm acclimation significantly increased the temperature at which acute warming of fish in normoxic water lost their righting reflex, increasing CT_max_ from 29.0 ± 0.4°C for 15°C-acclimated fish to 31.1°C for 23°C-acclimated fish (F_5,53_ = 95.5, *P* < 0.0001). In contrast to the other physiological impairments seen at the 25°C-acclimation temperature, CT_max_ was unchanged (31.2°C; Fig. 3B).

**Figure 4:**
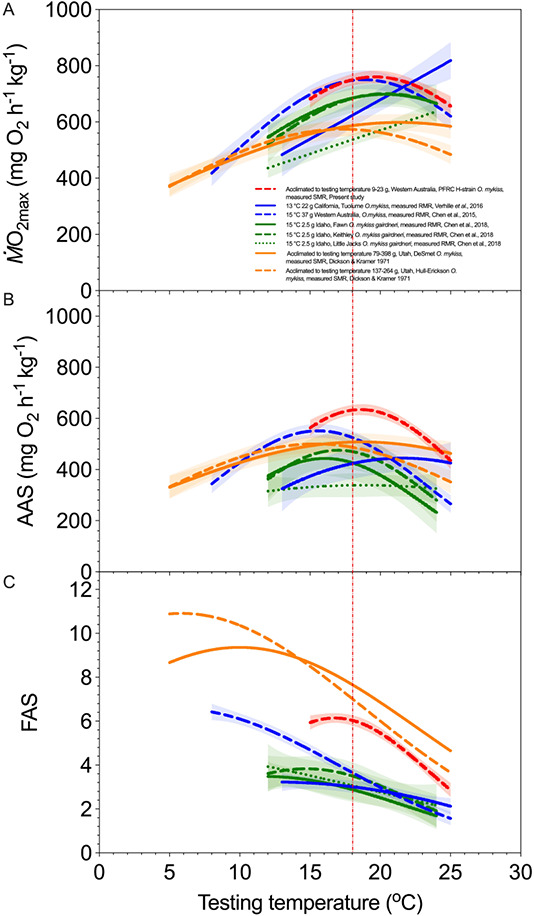
Maximum oxygen uptake (*Ṁ*O_2max_), absolute aerobic scope (AAS) and factorial aerobic scope (FAS) as a function of acclimation temperature. The data are synthesized from literature for geographically distant rainbow trout (*O. mykiss*) strains. *Ṁ*O_2max_; (**A**), AAS (**B**) and FAS (**C**) over a range of testing temperatures in various studies on rainbow trout (*O. mykiss*) strains. The data are presented in either Gaussian or linear regressions models as mean ± 95% C.I. Figure legends state the acclimation temperature, body mass, location, strains, original studies and whether standard metabolic rate (SMR) or resting metabolic rate (RMR) are measured. The studies had different mass, testing protocols and analytical techniques, as well as acclimation temperatures (indicated), but not all of the differences in the curves can be attributed to these. A red vertical dash line marks 18°C as the 7-day average of the daily maxima criterion for the management of rainbow trout habitat in the Pacific Northwest (U.S. Environmental Protection Agency, 2003). Rainbow trout (*O. mykiss*) and redband trout (*O. mykiss gairdneri*) are in blue and green, respectively, where the aerobic capacity thermal performance curves are measured by acute exposure. The performance curves measured by the previous acclimation study are in orange, and the data of the present acclimation study are in red. Patterns of lines differentiate the strains. The range of the curve is constrained by testing temperature ranges. This is modified from McKenzie *et al*. (2021) by adding data from Dickson and Kramer (1971; a 95% CI for FAS was unavailable) and excluding data from Poletto *et al*. (2017).

## Discussion

Our discovery of an unexpectedly high thermal acclimation potential for the H-strain of rainbow trout found in Western Australia, as compared with other studied strains of rainbow trout (Fig. 4), has an important ecological implication. It suggests that sufficient standing genetic variation could exist naturally within existing rainbow trout populations to provide sufficient intra-specific thermal acclimation potential to deal with a certain degree of global warming. Furthermore, by examining a broad range of thermal performance curves for a wide range of physiological function, we provided insights into the physiological mechanisms that underlie transition temperatures, thermal optima and tipping points (when a performance metric no longer increased or decreased). Importantly, and despite performing these physiological studies at a geographically remote field site, the accuracy of interpolating upper thermal tipping points was improved by using 2°C increments in acclimation temperature rather than more typical increments of 3–5°C in previous studies and focusing on temperatures at 15°C and beyond. Indeed, our results, which were obtained at two levels of biological organization, consistently showed upper thermal tipping points at acclimation temperatures of 21°C and 23°C.

Intra-specific differences in thermal acclimation potential for growth performance are already known for rainbow trout. For example, Myrick and Cech (2000) compared two Californian strains of rainbow trout, the Mount Shasta and Eagle Lake strains. Peak growth rate occurred between 19°C and 22°C for the Mount Shasta strain, while the Eagle Lake strain grew best at a cooler 19°C-acclimation temperature. Nevertheless, the Mount Shasta strain slowed growth at a 22°C-acclimation temperature and ceased growing and lost weight at the 25°C-acclimation temperature. The Eagle Lake strain resembled other rainbow trout strains that have peak growth performance at acclimation temperatures of 15.0–18.6°C (Hokanson *et al.,* 1977), the data used by the USEPA for rainbow trout management in the Pacific Northwest (U.S. Environmental Protection Agency, 2003). The H-strain of rainbow trout has a higher thermal growth performance than other strains thus far examined by still growing significantly at an acclimation temperature of 25°C (a 1.7-fold increase over 4 weeks), even though growth performance started to fall off at an acclimation temperature of ≥21°C.

Suppression of appetite at supra-optimal temperatures is a well-established phenomenon in fishes (Jobling, 1981; Myrick and Cech, 2000). The decline in growth potential when the H-strain was acclimated 25°C was certainly associated with a suppression of appetite (the lowest feed intake) and the lowest feed conversion efficiency (the highest FCR, which was optimal between 17°C and 23°C). An aerobic scope protection hypothesis has been advanced to explain appetite suppression at high temperature (Jutfelt *et al*., 2021), one that proposes that meal size is reduced to limit postprandial SDA and ‘protect’ residual aerobic scope. But how fishes might achieve and regulate such a protection is unknown. Our data SDA determinations provide new insights. As expected, *Ṁ*O_2_ peaked during digestion (Fu *et al.,* 2007; Eliason *et al.,* 2008; Pang *et al.,* 2011; Eliason and Farrell, 2014) and SDA_peak_ increased significantly as a function of acclimation temperature, even at 25°C. SMR also increased with acclimation temperature, as expected (e.g. Sandblom *et al.,* 2014; Claireaux *et al*., 2000), which would add to the SDA during digestion. To remove this effect, we calculated net SDA_peak_, which still increased with acclimation temperature up to 25°C. Thus, neither SDA_peak_ nor net SDA_peak_ appeared to have a ceiling at the supra-optimal acclimation temperature of 25°C, as might be expected for the aerobic scope protection hypothesis. Instead, we found considerable appetite suppression in 25°C-acclimated rainbow trout, an ample aerobic scope to buffer SDA_peak_ (which was about half of FAS), but costly food processing of a reduced meal size. Moreover, appetite suppression was triggered at acclimation temperatures cooler than 25°C when FAS was higher and SDA_peak_ was lower. SDA duration was shortest at acclimation temperatures between 19°C and 25°C, and SDA magnitude was lowest at 21°C. Consequently, we compared the ratio of SDA_peak_ and SMR with the ratio of *Ṁ*O_2max_ and SMR (i.e. FAS) for each acclimation temperature (this comparison was possible because SMR for the feeding and aerobic scope trials were indistinguishable). SDA_peak_/SMR was almost independent of acclimation temperature and, importantly, was never more than twice SMR because the increases in SDA_peak_ and SMR with acclimation temperature closely matched each other. How such a balance of metabolic rates might be regulated is unclear from the present work, but a similar discovery was made in lionfish (*Pterois* spp.) (Steell *et al.,* 2019). Thus, while our findings are consistent with the aerobic scope protection hypothesis in that appetite falls off seeming in parallel with FAS, the entire FAS was never fully exploited by SDA_peak_ and a variable aerobic scope buffer exists for different levels of appetite suppression as a function of acclimation temperature. How these buffer capacities might be regulated and what determines their magnitude will require further study, as will our discovery that digestion of a small meal became less efficient at a supra-optimal acclimation temperature.

Work with low-altitude or high-altitude populations of killifish (genus *Aphysosemion*) in equatorial West Africa previously showed SDA performance declined at acclimation temperatures outside of the ecologically relevant temperature (25°C) for this genus (McKenzie *et al*., 2013). Specifically, the low-altitude population processed a meal much faster than a high-altitude population when tested at the supra-optimal acclimation temperature of 28°C. At a sub-optimal acclimation temperature of 19°C, the converse was true. However, the reduced digestive performance in killifish was not associated with any change in SDA_peak_ (McKenzie *et al*., 2013), unlike the present study where acclimation temperature affected the speed with which the H-strain of rainbow trout processed a meal, with the nadir in SDA duration occurring at acclimation temperatures of 21°C and 23°C.

The unusually high upper thermal acclimation potential of H-strain rainbow trout is highlighted by comparing our *Ṁ*O_2max_ and AAS curves with literature values for this genus (Fig. 4). (Note: Our AAS for the H-strain rainbow trout at an acclimation temperature of 15°C compares favourably an early measurement at this acclimation temperature: 544 mg O_2_ kg^−1^ h^−1^ for Chen *et al.,* 2015 and 558 mg O_2_ kg^−1^ h^−1^ for the present study.) Our results reflect that the H-strain of rainbow trout has the potential to acclimate to temperatures more comparable with those for the redband rainbow trout, a subspecies of rainbow trout that successfully adapted to desert habitats after becoming geographically isolated from the Columbia River in Idaho and Oregon, USA. Redband rainbow trout routinely experience highly variable and hot summer temperatures, e.g. 19–29°C for 12-mile Creek (Rodnick *et al.,* 2004). Furthermore, the H-strain of rainbow trout has a higher aerobic performance and thermal optimum for *Ṁ*O_2max_ compared with two southern Utah strains of rainbow trout (Fig. 4; Hull-Erickson strain and DeSmet strain; Dickson and Kramer, 1971). Furthermore, the aerobic benefit of the H-strain’s ability to warm acclimate to 23°C can now be quantified by comparing the *Ṁ*O_2max_ after acute warming to 23°C (Chen *et al.,* 2015) with that after acclimation to 23°C. Acutely warming to 23°C resulted in a 30% lower *Ṁ*O_2max_ and a 19% lower AAS than measured after acclimation to 23°C. Thus, the remarkable phenotypic plasticity for thermal acclimation of the H-strain rainbow trout provides a clear and substantial benefit to aerobic capacity at an acclimation temperature of 23°C. While another Californian population of rainbow trout can maintain 95% of its peak aerobic capacity when acutely warmed to 24.5°C (Verhille *et al.,* 2016), its thermal acclimation potential is unmeasured. A pressing and unanswered question, therefore, is whether or not sufficient standing genetic variation exists within natural rainbow trout populations to acclimate to temperatures well above 20°C and deal with a certain degree of global warming. The Californian founder population of the H-strain and the redband trout certainly do.

The primary cardiac response of a fish to acute warming is to increase *f*_H_ (e.g. Sandblom and Axelsson, 2007; Steinhausen *et al.,* 2008; Ekstrom *et al*., 2016), which has an upper limit (Farrell, 2016; Gilbert *et al.,* 2019). Indeed, increasing *f*_H_ is the primary mechanism to meet an inexorable increase in tissue oxygen demand during acute warming when the mitochondrial efficiency of using oxygen to generate ATP sometimes decreases (Little *et al.,* 2020). Even when fish are swimming, acute warming increases *f*_H_ up to a peak value (Steinhausen *et al.,* 2008; Eliason *et al.,* 2011, 2013). Our data for the effect of acute warming on *f*_Hmax_ are entirely consistent with this literature. Furthermore, warm acclimation of the H-strain helped retain a scope to increase *f*_H_ during acute warming, as shown previously for other fishes, which lowered intrinsic *f*_H_ and increased T_peak_, T_arr_ and peak *f*_H_ (Farrell, 1991, 2016; Farrell and Smith, 2017). Warm acclimation of the H-strain of rainbow trout reset their *f*_Hmax_ (22-bpm lower in 25°C-acclimated than the 15-acclimated fish when tested at 12°C), increased their T_peak_ and T_arr_ (by 2.7°C and by 3.4°C, respectively, for 15°C- and 23°C-acclimated fish) but did not increase their peak *f*_Hmax_. Similar to the earlier acute warming study of the H-strain rainbow trout acclimated to 15°C (Chen *et al.,* 2015), our T_peak_ for *f*_Hmax_ for 15°C-acclimated fish (24.7°C) is similar to their value (23.5–24.0°C), as is the T_arr_ value (25.5°C vs 24.7–25.7°C). Peak *f*_Hmax_ (170–180 bpm) of the H-strain stood out compared with lower values for *f*_Hmax_ in other salmonids (e.g. Steinhausen *et al.,* 2008; Eliason *et al.,* 2011, 2013; Gilbert *et al.,* 2019) including rainbow trout (≤130 bpm; Wood *et al.,* 1979). The mechanism behind the faster heartbeat of the H-strain may be an important factor for their high thermal tolerance given that excessive warming is thought to eventually trigger conduction failure and cardiac arrhythmias in fish (Anttila *et al.,* 2014; Farrell, 2016; Vornanen, 2017).

Any cardiac benefits of warm acclimation were clearly lost for the H-strain of rainbow trout acclimated to 25°C because peak *f*_Hmax_ (151 ± 6 bpm), T_peak_ (26.2 ± 0.8°C) and T_arr_ (27.2 ± 0.6°C) all decreased significantly. Indeed, their scope to increase *f*_Hmax_ during acute warming was small given that peak *f*_Hmax_ was reached at 26.2°C. In view of this and because resting and maximum *f*_H_ tend to converge at warm temperatures in trout and salmon (Steinhausen *et al.,* 2008; Farrell, 2009; Eliason *et al.,* 2011; Eliason *et al.,* 2013), we propose a novel mechanism to explain both the appetite suppression and post-handling mortality in 25°C-acclimated H-strain trout that we observed. Increasing routine *f*_H_ is the primary cardiac response of fishes to meet the elevated *Ṁ*O_2_ associated with SDA and dealing with handling stress (Clark *et al.,* 2008; Eliason *et al.,* 2008; Chen *et al.,* 2018). Clearly, the capacity to mount such increases became very limited at the 25°C acclimation temperature. Lacking a capacity to increase *f*_H_ for digestion certainly aligns with the hypothesis purporting that cardiac function is a potential weak link for upper thermal tolerance of fishes (Wang and Overgaard, 2007; Pörtner and Farrell, 2008; Farrell, 2009). This mechanism would also align with the aerobic scope protection hypothesis (tissue oxygen extraction and cardiac stroke volume can still potentially increase somewhat).

CT_max_ is likely the most commonly used indicator of upper thermal tolerance for fish and rainbow trout as a species appear to have an upper ceiling for its upper thermal tolerance as measured by CT_max_. Our CT_max_ value for 15°C-acclimated fish (29.0°C) compares well with an earlier determination for the H-strain acclimated to 15°C (28.1–29.7°C; Chen *et al.,* 2015) and for a 15°C-acclimated Japanese strain of rainbow trout, which has been intensely selected for upper thermal tolerance over multiple generations and originally from California (29.7°C; Ineno *et al.,* 2005). The CT_max_ for redband trout (28.8–29.8°C; Rodnick *et al.,* 2004; Chen *et al.,* 2018) is close to the upper daily water temperature recorded for their stream habitat (Rodnick *et al.,* 2004). In contrast, CT_max_ values ranging from 21°C to 26.5°C are much lower for steelhead trout (*O. mykiss*) from the Columbia River and North Santiam River (Oregon) (Coutant, 1970; Redding and Schreck, 1979) than the 25°C-acclimated H-strain rainbow trout, which maintained CT_max_ around 31°C. Yet, this maintained upper thermal tolerance clearly contrasted with other physiological performance variables that had failed at this acclimation temperature, i.e. growth, appetite, FCR, the oxygen cost of digesting a meal and an increasing heart rate with acute warming. Likewise, the similar CT_max_ values for the Californian Eagle Lake (30.6°C) and Mount Shasta (30.0°C) strains of rainbow trout do not reflect intra-specific differences in their thermal growth performance curves (Myrick and Cech, 2000). Therefore, the merit of CT_max_ as a useful ecological indicator of upper thermal tipping points for life-supporting physiological functions must be questioned, despite the ease with which the measurement can be made. This concern is readily seen by using a thermal tolerance polygon (Fry, 1971) to directly compare CT_max,_ T_arr_ and T_peak_ (Fig. 3B; Table S2), physiological indices that were determined with a similar acute warming protocol. Independent of the acclimation temperature, T_arr_ and T_peak_ are consistently lower than CT_max_ (minimally by 2°C for T_arr_ and even more for T_peak_), but the magnitude of this difference varies with acclimation temperature, being greatest amount at 15°C and 25°C, e.g. CT_max_ was 5°C higher than T_peak_ (26.2°C) at 25°C. This comparison provides compelling evidence that limitations to increasing *f*_H_ during acute warming can occur well below CT_max_ and that the faltering ability of the heart to deliver oxygen at different acclimation temperatures is not reliably predicted by CT_max_ for the H-strain of rainbow trout.

In conclusion, our upper thermal performance curves for the H-strain rainbow trout suggest that its Californian founder population had a suite of genes that were preferentially selected over many generations of inbreeding in an Australian desert climate to generate a strain with an unusually high upper thermal acclimation potential. Indeed, the H-strain seems most similar to a cousin, the redband trout, which has naturally adapted to desert streams that can reach 29°C. Despite this encouraging discovery for those with conservation concerns for rainbow trout and other fish species, a 25°C-acclimation temperature may not be sustainable for the H-strain rainbow trout given their slow growth rate, reduced appetite and aerobic scope, inefficient food conversion and processing, limited scope to increase *f*_H_ during warming and limited ability to tolerate handling stress. As seen for rainbow trout populations introduced to Japan and Argentina (Ineno *et al.,* 2005; Crichigno *et al.,* 2018) and much like the Swedish perch in the warm-water discharge enclosure of a nuclear power plant, which have a higher thermal tolerance than the endemic and founding perch population (Sandblom *et al.,* 2016), the standing variation of a founder population may be an undiscovered but crucial source for biogeographic redistributions of certain fish species. Intra-specific differences in upper thermal performance now need to be sought in populations living at the southern end of their biogeographic range and, if discovered, will need to be considered when managing and modelling fish habitats for conservation purposes. Whether convergent evolution of high upper thermal performance respiratory phenotypes has a common or different suite of physiological mechanisms remains to be explored.

## Funding

This project was supported by funding from Natural Sciences and Engineering Research Council of Canada to A.P.F., who holds a Canada Research Chair, and Mitacs Globalink Research funding to O.A.A. Travelling Fellowships from the Company of Biologists subsidized logistic costs of Y.Z. Y.Z. held Elizabeth R Howland Fellowship & Pei-Huang Tung and Tan-Wen Tung Graduate Fellowship.

## Author Contributions

O.A.A. helped design the project, performed all the experimental work, performed data analysis, wrote the initial draft of the manuscript and performed subsequent edits. C.S.L., M.S. and A.P.F. conceived and designed the experiments and contributed to the writing of the manuscript. Y.Z. helped with setting up the experimental work, contributed to the data analysis, synthesized the thermal performance curves of aerobic capacity and assisted in the writing of the manuscript. M.J.H.G. contributed to the cardiac methodology, data analysis, editing of the manuscript.

## Competing Interest

The authors declare no competing interests.

## Supplementary Material

Supplemental_Tables_coab101

## References

[ref1] Anttila K, Courturier CS, Øverli Ø, Johnsen A, Marthinsen G, Nilsson GE, Farrell AP (2014) Atlantic salmon show capability for cardiac acclimation to warm temperatures. Nat Commun 5: 1–6. 10.1038/ncomms5252 24957572

[ref2] Becker CD, Wolford MG (1980) Thermal Resistance of Juvenile Salmonids Sublethally Exposed to Nickel, Determined by the Critical Thermal Maximum Method. Environmental Pollution (Barking, Essex: 1987) 21: 181–189.

[ref3] Beitinger T, Bennett W, McCauley R (2000) Temperature tolerances of North American freshwater fishes exposed to dynamic changes in temperature. Environ Biol Fishes 58: 237–275.

[ref4] Casselman MT, Anttila K, Farrell AP (2012) Using maximum heart rate as a rapid screening tool to determine optimum temperature for aerobic scope in Pacific salmon *Oncorhynchus spp*. J Fish Biol 80: 358–377. 22268435 10.1111/j.1095-8649.2011.03182.x

[ref5] Chabot D, Koenker R, Farrell AP (2016a) The measurement of specific dynamic action in fishes: measuring SDA in fishes. J Fish Biol 88: 152–172. 26768974 10.1111/jfb.12836

[ref6] Chabot D, Steffensen JF, Farrell AP (2016b) The determination of standard metabolic rate in fishes: measuring SMR in fishes. J Fish Biol 88: 81–121. 26768973 10.1111/jfb.12845

[ref7] Chen Z, Farrell AP, Matala A, Narum SR (2018) Mechanisms of thermal adaptation and evolutionary potential of conspecific populations to changing environments. Mol Ecol 27: 659–674. 29290103 10.1111/mec.14475

[ref8] Chen Z et al. (2015) Selection for upper thermal tolerance in rainbow trout (*Oncorhynchus mykiss* Walbaum). J Exp Biol 218: 803–812. 25573825 10.1242/jeb.113993

[ref9] Cheung WWL et al. (2006) Intraspecific variation in thermal tolerance and heat shock protein gene expression in common killifish, *Fundulus heteroclitus*. J Exp Biol 209: 2859–2872. 16857869 10.1242/jeb.02260

[ref10] Christensen EAF, Svendsen MBS, Steffensen JF (2020) The combined effect of body size and temperature on oxygen consumption rates and the size-dependency of preferred temperature in European perch *Perca fluviatilis*. J Fish Biol 97: 794–803. 32557687 10.1111/jfb.14435

[ref11] Claireaux G, Chabot D (2016) Responses by fishes to environmental hypoxia: integration through Fry’s concept of aerobic metabolic scope: hypoxia and fry’s paradigm of aerobic scope. J Fish Biol 88: 232–251. 26768976 10.1111/jfb.12833

[ref12] Claireaux G, Webber DM, Lagardère J-P, Kerr SR (2000) Influence of water temperature and oxygenation on the aerobic metabolic scope of Atlantic cod (*Gadus morhua*). J Sea Res 44: 257–265.

[ref13] Claireaux G et al. (2005) Linking swimming performance, cardiac pumping ability and cardiac anatomy in rainbow trout. J Exp Biol 208: 1775–1784. 15879059 10.1242/jeb.01587

[ref14] Clark TD, Sandblom E, Cox GK, Hinch SG, Farrell AP (2008) Circulatory limits to oxygen supply during an acute temperature increase in the Chinook salmon (*Oncorhynchus tshawytscha*). Am J Physiol-Regul Integr Comp Physiol 295: R1631–R1639. 18768764 10.1152/ajpregu.90461.2008

[ref15] Clark TD et al. (2005) Factorial aerobic scope is independent of temperature and primarily modulated by heart rate in exercising Murray cod (*Maccullochella peelii peelii*). Physiol Biochem Zool 78: 347–355. 15887081 10.1086/430034

[ref16] Coutant CC (1970) Thermal resistance of adult coho (*Oncorhynchus kisutch*) and jack chinook (*O. tshawytscha*) salmon, and adult steelhead trout (*Salmo gairdneri*) from the Columbia river. Pacific Northwest Lab., Richland, WA, USA. 10.2172/4107005.

[ref17] Crichigno SA et al. (2018) Rainbow trout adaptation to a warmer Patagonia and its potential to increase temperature tolerance in cultured stocks. Aquac Rep 9: 82–88.

[ref18] Davenport J, Sayer MDJ (1993) Physiological determinants of distribution in fish. J. Fish Biol 43: 121–145.

[ref19] Dickson IW, Kramer RH (1971) Factors influencing scope for activity and active and standard metabolism of rainbow trout (*Salmo gairdneri*). J Fish Res Bd Can 28: 587–596.

[ref70] Ekström A, Brijs J, Clark TD, Gräns A, Jutfelt F, Sandblom M (2016) Cardiac oxygen limitation during an acute thermal challenge in the European perch: effects of chronic environmental warming and experimental hyperoxia. Am J Physiol Regul Integr Comp Physiol 311: R440–R449. 27280433 10.1152/ajpregu.00530.2015

[ref20] Eliason EJ, Anttila K (2017) Temperature and the cardiovascular system. The Cardiovascular System: Development, Plasticity and Physiological Responses vol. 36. Academic Press, Elsevier, Cambridge, Massachusette, United States, pp. 235–297.

[ref21] Eliason EJ, Clark TD, Hinch SG, Farrell AP (2013) Cardiorespiratory collapse at high temperature in swimming adult sockeye salmon. Conserv Physiol 1. 10.1093/conphys/cot008 PMC4732444 27293592

[ref21a] Eliason EJ, Farrell AP (2014) Effect of hypoxia on specific dynamic action and postprandial cardiovascular physiology in rainbow trout (Oncorhynchus mykiss). Comparative Biochemistry and Physiology Part A: Molecular & Integrative Physiology. 171, 44–50. 10.1016/j.cbpa.2014.01.021 24534150

[ref22] Eliason EJ, Higgs DA, Farrell AP (2008) Postprandial gastrointestinal blood flow, oxygen consumption and heart rate in rainbow trout (*Oncorhynchus mykiss*). Comp Biochem Physiol A Mol Integr Physiol 149: 380–388. 18308602 10.1016/j.cbpa.2008.01.033

[ref23] Eliason EJ et al. (2011) Differences in thermal tolerance among sockeye salmon populations. Science 332: 109–112. 21454790 10.1126/science.1199158

[ref68] Fangue NA, Hofmeister M, Schulte PM (2006) Intraspecific variation in thermal tolerance and heat shock protein gene expression in common killifish, Fundulus heteroclitus. J Exp Biol 209: 2859–2872. 16857869 10.1242/jeb.02260

[ref24] Fangue NA, Hofmeister M, Schulte PM (2014) Mechanisms of reef coral resistance to future climate change. Science 344: 895–898. 24762535 10.1126/science.1251336

[ref25] Farrell AP (1991) From hagfish to tuna: a perspective on cardiac function in fish. Physiol Zool 64: 1137–1164.

[ref26] Farrell AP (2009) Environment, antecedents and climate change: lessons from the study of temperature physiology and river migration of salmonids. J Exp Biol 212: 3771–3780. 19915118 10.1242/jeb.023671

[ref27] Farrell AP (2016) Pragmatic perspective on aerobic scope: peaking, plummeting, pejus and apportioning. J Fish Biol 88: 322–343. 26592201 10.1111/jfb.12789

[ref69] Farrell AP, Smith F (2017) Cardiac form, function and physiology. In Fish physiology vol. 36. Academic Press, pp. 155–264.

[ref28] Fry FEJ (1971) The effect of environmental factors on the physiology of fish. In WS Hoar, DJ Randall, eds, Fish Physiology, Vol. 6. Academic Press, pp. 1–98

[ref29] Fu SJ, Cao Z-D, Peng JL (2007) Effect of feeding and fasting on excess post-exercise oxygen consumption in juvenile southern catfish (*Silurus meridionalis* Chen). Comp Biochem Physiol A Mol Integr Physiol 146: 435–439. 17251045 10.1016/j.cbpa.2006.12.002

[ref30] Gilbert MJH, Rani V, McKenzie SM, Farrell AP (2019) Autonomic cardiac regulation facilitates acute heat tolerance in rainbow trout: in situ and in vivo support. J Exp Biol 222: jeb194365. 31015284 10.1242/jeb.194365

[ref31] Grabowski TB, Young SP, Libungan LA, Steinarsson A, Marteinsdóttir G (2009) Evidence of phenotypic plasticity and local adaption in metabolic rates between components of the Icelandic cod (*Gadus morhua L*.) stock. Environ Biol Fish 86: 361–370.

[ref32] Hokanson KEF, Kleiner CF, Thorslund TW (1977) Effects of constant temperatures and diel temperature fluctuations on specific growth and mortality rates and yield of juvenile rainbow trout, *Salmo gairdneri*. J Fish Res Board Can 34: 639–648.

[ref33] Ineno T, Tsuchida S, Kanda M, Watabe S (2005) Thermal tolerance of a rainbow trout *Oncorhynchus mykiss* strain selected by high-temperature breeding. Fish Sci 71: 767–775.

[ref34] Jobling M (1981) The influences of feeding on the metabolic rate of fishes: a short review. J Fish Biol 18: 385–400.

[ref35] Jutfelt F, Norin T, Åsheim ER, Rowsey LE, Andreassen AH, Morgan R, Clark TD, Speers-Roesch B (2021) Aerobic scope protection reduces ectotherm growth under warming. Funct Ecol. 10.1111/1365-2435.13811.

[ref36] Lefevre S, McKenzie DJ, Nilsson GE (2017) Models projecting the fate of fish populations under climate change need to be based on valid physiological mechanisms. Glob Chang Biol 23: 3449–3459. 28168760 10.1111/gcb.13652

[ref37] Little AG, Loughland I, Seebacher F (2020) What do warming waters mean for fish physiology and fisheries? J Fish Biol 97: 328–340. 32441327 10.1111/jfb.14402

[ref38] MacCrimmon HR (1971) World distribution of rainbow trout (*Salmo gairdneri*). J Fish Res Bd Can 28: 663–704.

[ref39] McKenzie DJ, Estivales G, Svendsen JC, Steffensen JF, Agne’se, J-F. (2013) Local adaptation to altitude underlies divergent thermal physiology in tropical killifishes of the genus Aphyosemion. PLoS One 8: e54345. 10.1371/journal.pone.0054345. 23349857 PMC3551936

[ref40] McKenzie DJ, Zhang Y, Eliason EJ, Schulte PM, Claireaux G, Blasco FR, Nati JJH, Farrell AP (2021) Intraspecific variation in tolerance of warming in fishes. J Fish Biol 98: 1536–1555. 10.1111/jfb.14620. 33216368

[ref41] Molony B (2001) Environmental Requirements and Tolerances of Rainbow Trout (Oncorhynchus mykiss) and Brown Trout (Salmo Trutta) with Special Reference to Western Australia: A Review. Vol. 130. Department of Fisheries, Government of Western Australia.

[ref42] Molony BW, Church AR, Maguire G, B. (2004) A comparison of the heat tolerance and growth of a selected and non-selected line of rainbow trout, *Oncorhynchus mykiss*, in Western Australia. Aquaculture 241: 655–665.

[ref43] Myrick CA, Cech JJ (2000) Temperature influences on California rainbow trout physiological performance. Fish Physiol Biochem 22: 245–254.

[ref44] Myrick CA, Cech JJ (2004) Temperature effects on juvenile anadromous salmonids in California’s central valley: what don’t we know? Rev Fish Biol Fish 14: 113–123.

[ref45] Pang X, Cao Z-D, Fu SJ (2011) The effects of temperature on metabolic interaction between digestion and locomotion in juveniles of three cyprinid fish (*Carassius auratus*, *Cyprinus carpio* and *Spinibarbus sinensis*). Comp Biochem Physiol A Mol Integr Physiol 159: 253–260. 21440661 10.1016/j.cbpa.2011.03.013

[ref46] Pereira HM et al. (2010) Scenarios for global biodiversity in the 21st century. Science 330: 1496–1501. 20978282 10.1126/science.1196624

[ref47a] Poletto JB, Cocherell DE, Baird SE, Nguyen TX, Cabrera-Stagno V, Farrell AP, Fangue NA (2017) Unusual aerobic performance at high temperatures in juvenile Chinook salmon, Oncorhynchus tshawytscha. Conservation physiology 5(1) cow067. 28078086 10.1093/conphys/cow067 PMC5216678

[ref47] Pörtner HO, Farrell AP (2008) Physiology and climate change. Science, 322: 690–692. 18974339 10.1126/science.1163156

[ref48] Redding JM, Schreck CB (1979) Possible adaptive significance of certain enzyme polymorphisms in steelhead trout (*Salmo gairdneri*). J Fish Res Board Can 36: 544–551.

[ref49] Reite OB, Maloiy GMO, Aasehaug B (1974) pH, salinity and temperature tolerance of Lake Magadi tilapia. Nature 247: 315–315.

[ref50] Rodnick KJ et al. (2004) Thermal tolerance and metabolic physiology among redband trout populations in south-eastern Oregon. J Fish Biol 64: 310–335.

[ref51] Sandblom E, Axelsson M (2007) Venous hemodynamic responses to acute temperature increase in the rainbow trout (*Oncorhynchus mykiss*). Am J Physiol-Regul Integr Comp Physiol 292: R2292–R2298. 17322113 10.1152/ajpregu.00884.2006

[ref52] Sandblom E, Gräns A, Axelsson M, Seth H (2014) Temperature acclimation rate of aerobic scope and feeding metabolism in fishes: implications in a thermally extreme future. Proc R Soc B Biol Sci 281: 20141490. 10.1098/rspb.2014.1490 PMC4211447 25232133

[ref53] Sandblom E et al. (2016) Physiological constraints to climate warming in fish follow principles of plastic floors and concrete ceilings. Nat Commun 7: 1–8. 10.1038/ncomms11447 PMC4873662 27186890

[ref54] Schurmann H, Steffensen JF (1997) Effects of temperature, hypoxia and activity on the metabolism of juvenile Atlantic cod. J Fish Biol 50: 1166–1180.

[ref55] Steell SC, Van Leeuwen TE, Brownscombe JW, Cooke SJ, Eliason EJ (2019) An appetite for invasion: digestive physiology, thermal performance and food intake in lionfish (*Pterois* spp.). J Exp Biol 222: jeb209437. 31527176 10.1242/jeb.209437

[ref56] Steinhausen MF, Sandblom E, Eliason EJ, Verhille C, Farrell AP (2008) The effect of acute temperature increases on the cardiorespiratory performance of resting and swimming sockeye salmon (*Oncorhynchus nerka*). J Exp Biol 211: 3915–3926. 19043063 10.1242/jeb.019281

[ref57] U.S. Environmental Protection Agency (2003) EPA Region 10 Guidance for Pacific Northwest State and Tribal Temperature Water Quality Standards, EPA 910-B-03-002. Region 10 Office of Water, Seattle, WA.

[ref58] Verhille CE, English KK, Cocherell DE, Farrell AP, Fangue NA (2016) High thermal tolerance of a rainbow trout population near its southern range limit suggests local thermal adjustment. Conserv Physiol 4: cow057. 27957333 10.1093/conphys/cow057 PMC5146681

[ref59] Vornanen M (2017) Electrical excitability of the fish heart and its autonomic regulation. In AK Gamperl, TE Gillis, AP Farrell, CJ Brauner, eds, Fish Physiology, Vol. 36. Academic Press, pp. 99–153

[ref60] Wang T, Overgaard J (2007) The heartbreak of adapting to global warming. Science 315: 49–50. 17204631 10.1126/science.1137359

[ref61] Ward RD, Jorstad KE, Maguire GB (2003) Microsatellite diversity in rainbow trout *(Oncorhynchus mykiss)* introduced to Western Australia. Aquaculture 219: 169–179.

[ref62] Wood CM, Pieprzak P, Trott JN (1979) The influence of temperature and anaemia on the adrenergic and cholinergic mechanisms controlling heart rate in the rainbow trout. Can J Zool 57: 2440–2447.

[ref63] Yu D, Zhang Z, Shen Z, Zhang C, Liu H (2018) Regional differences in thermal adaptation of a cold-water fish *Rhynchocypris oxycephalus* revealed by thermal tolerance and transcriptomic responses. Sci Rep 8: 1–11. 30076386 10.1038/s41598-018-30074-9 PMC6076256

[ref64] Zhang Y, Gilbert MJH, Farrell AP (2020) Measuring maximum oxygen uptake with an incremental swimming test and by chasing rainbow trout to exhaustion inside a respirometry chamber yields the same results. J Fish Biol. 97: 28–38. 32154581 10.1111/jfb.14311

[ref65] Zhang Y, Healy TM, Vandersteen W, Schulte PM, Farrell AP (2018) A rainbow trout *Oncorhynchus mykiss* strain with higher aerobic scope in normoxia also has superior tolerance of hypoxia. J Fish Biol 92: 487–503. 29431223 10.1111/jfb.13530

[ref66] Zhang Y, Mauduit F, Farrell AP, Chabot D, Ollivier H, Rio-Cabello A, Floch SL, Claireaux G (2017) Exposure of European sea bass (*Dicentrarchus labrax*) to chemically dispersed oil has a chronic residual effect on hypoxia tolerance but not aerobic scope. Aquat Toxicol 191: 95–104. 28806602 10.1016/j.aquatox.2017.07.020

[ref67] Zhang Y, Timmerhaus G, Anttila K, Mauduit F, Jørgensen SM, Kristensen T, Claireaux G, Takle H, Farrell AP (2016) Domestication compromises athleticism and respiratory plasticity in response to aerobic exercise training in Atlantic salmon (*Salmo salar*). Aquaculture 463: 79–88.

