# Supplementary material for: An unusually high upper thermal acclimation potential for rainbow trout
Source: Conserv Physiol. 2022 Jan 15;10(1):coab101. doi: 10.1093/conphys/coab101 (PMC9040278; doi:10.1093/conphys/coab101)
Supplement: Supplemental_Tables_coab101 [file supplemental_tables_coab101.docx]

Table S1. Body mass (g) and number (N) of the H-strain of rainbow trout (*Oncorhynchus mykiss*) used in each of the experimental trials that measured aerobic scope, specific dynamic action (SDA), critical thermal maximum (CT_max_) and maximum heart rate (*f*_Hmax_). Values are presented as mean ± sem and different letter subscripts indicate significant differences (p-value < 0.05) among the temperature acclimation groups.

| **Temperature acclimation group (˚C)** | **Aerobic scope** | | **SDA** | | **CT_max_** | | ***f*_Hmax_** | |
| --- | --- | --- | --- | --- | --- | --- | --- | --- |
|  | **N** | **Mass (g)** | **N** | **Mass (g)** | **N** | **Mass (g)** |  | **Mass (g)** |
| 15 | 16 | 18.5±0.6^a^ | 15 | 17.1±0.7 | 10 | 18.4±1.1^a^ | 12 | 18.7±0.6^a^ |
| 17 | 14 | 18.5±0.7^a^ | 10 | 17.0±0.9 | 10 | 17.5±0.9^a^ | 11 | 18.5±0.8^a^ |
| 19 | 15 | 17.3±0.9^a^ | 14 | 17.4±0.7 | 10 | 17.6±1.0^a^ | 10 | 17.4±1.0^b^ |
| 21 | 15 | 14.2±0.5^b^ | 13 | 14.9±0.4 | 10 | 15.9±1.0^a^ | 9 | 14.2±0.7^c^ |
| 23 | 15 | 14.1±0.4^b^ | 14 | 15.9±0.4 | 10 | 18.5±1.0^a^ | 11 | 16.5±0.9^b^ |
| 25 | 10 | 13.5±0.8^b^ | 10 | 14.9±0.9 | 9 | 23.0±1.4^b^ | 10 | 17.2±0.8^b^ |

**Table S2.** Summary of statistics for the effect of acclimation temperature and week on body mass and fork length (a mixed effects analysis), as well as for the effect of acclimation temperature and week on appetite, and for thermal tolerance as a function of critical thermal maximum (CT_max_) and temperature of cardiac arrhythmia (T_arr_) (2-way ANOVA). A significant effect for the H-strain of rainbow trout (*Oncorhynchus mykiss*) is indicated by a bold p-value (< 0.05).

|  |  | **F-value** | **df** | **p** |
| --- | --- | --- | --- | --- |
| Body mass | Interaction | 91.89 | 10,23 | **<0.0001** |
|  | Week | 3690 | 1.20,14.86 | **<0.0001** |
|  | Temperature | 91.89 | 5,12 | **<0.0001** |
| Fork length | Interaction | 87.21 | 10,23 | **<0.0001** |
|  | Week | 4249 | 1.23,14.16 | **<0.0001** |
|  | Temperature | 55.63 | 5,12 | **<0.0001** |
| Appetite | Interaction | 25.15 | 10,36 | **<0.0001** |
|  | Week | 266.3 | 2,36 | **<0.0001** |
|  | Temperature | 168.1 | 5,36 | **<0.0001** |
| Thermal tolerance | Interaction | 2.126 | 5,109 | 0.0677 |
|  | CT_max_ vs. T_arr_ | 14.12 | 5,109 | **<0.0001** |
|  | Acclimation Temp | 213.2 | 5,109 | **<0.0001** |
|  |  |  |  |  |

**Table S3.** Summary of statistics for the effect of acclimation temperature on specific growth rate (SGR), feeding rate, oxygen uptake (SMR, *Ṁ*O_2max_, AAS, FAS), specific dynamic action (SMR, peak and net peak SDA, duration), hypoxia tolerance (P_crit_, C_crit_, ILOP, ILOC) whole-animal and cardiac thermal tolerance (CT_max_, T_arr­_, T_peak_) and maximum heart rate (peak *ƒ*_Hmax_, *ƒ*_Hmax_ at 15°C) (analysis of variance). A significant effect of acclimation temperature on the H-strain of rainbow trout (*Oncorhynchus mykiss*) is indicated by a bold p-value (< 0.05).

|  | **F-value** | **df** | **p** |
| --- | --- | --- | --- |
| SGR (% day^-1^) | 144.6 | 5,11 | **<0.0001** |
| Feeding rate (% day^-1^) | 57.9 | 5,11 | **<0.0001** |
|  |  |  |  |
| SMR | 32 | 5,79 | **<0.0001** |
| *Ṁ*O_2max_ | 5.3 | 5,79 | **0.0003** |
| AAS | 11.8 | 5,79 | **<0.0001** |
| FAS | 14.4 | 5,79 | **<0.0001** |
|  |  |  |  |
| SMR (SDA) | 34.3 | 5,71 | **<0.0001** |
| Peak SDA | 73.8 | 5,71 | **<0.0001** |
| Net Peak SDA | 16.7 | 5,71 | **<0.0001** |
| Duration | 7.9 | 5,71 | **<0.0001** |
|  |  |  |  |
| P_crit_ | 22.19 | 5,69 | **<0.0001** |
| C_crit_ | 11.58 | 5,69 | **<0.0001** |
| ILOP | 6.41 | 5,73 | **<0.0001** |
| ILOC | 1.91 | 5,73 | 0.10 |
|  |  |  |  |
| CT_max_ | 95.46 | 5,53 | **<0.0001** |
| T_arr_ | 6.29 | 5,56 | **0.0001** |
| T_peak_ | 5.33 | 5,56 | **0.0004** |
| peak *ƒ*_Hmax_ | 5.27 | 5,56 | **0.0005** |
| *ƒ*_Hmax_ at 15°C | 23.11 | 5,52 | **<0.0001** |
